# Porous Framework Materials for Bioimaging and Cancer Therapy

**DOI:** 10.3390/molecules28031360

**Published:** 2023-01-31

**Authors:** Meng Jin, Yingying Zhao, Zong-Jie Guan, Yu Fang

**Affiliations:** 1 State Key Laboratory for Chemo/Bio-Sensing and Chemometrics, College of Chemistry and Chemical Engineering, Hunan University, Changsha 410082, China; 2 Innovation Institute of Industrial Design and Machine Intelligence, Quanzhou-Hunan University, Quanzhou 362801, China

**Keywords:** cancer, bioimaging, photothermal therapy, chemodynamic therapy, photodynamic therapy, porous framework materials

## Abstract

Cancer remains one of the most pressing diseases in the world. Traditional treatments, including surgery, chemotherapy, and radiotherapy still show certain limitations. Recently, numerous cancer treatments have been proposed in combination with novel materials, such as photothermal therapy, chemodynamic therapy, immunotherapy, and a combination of therapeutic approaches. These new methods have shown significant advantages in reducing side effects and synergistically enhancing anti-cancer efficacy. In addition to the above approaches, early diagnosis and in situ monitoring of lesion areas are also important for reducing side effects and improving the success rate of cancer therapy. This depends on the decent use of bioimaging technology. In this review, we mainly summarize the recent advances in porous framework materials for bioimaging and cancer therapy. In addition, we present future challenges relating to bioimaging and cancer therapy based on porous framework materials.

## 1. Introduction

Cancer, one of the major causes resulting in human death, has caused significant economic losses and serious health problems every year [1]. Great efforts have been made in improving cancer treatment conditions, however, the total number of cancer deaths remains high per year [2]. Currently, surgery, chemotherapy, and radiotherapy are the three main traditional methods for cancer treatment [3]. Nevertheless, these methods have some limitations such as severe side effects and recurrence [4]. In addition to the above traditional treatments, several new cancer treatment methods based on nanomaterials have emerged in recent years [5,6,7,8,9,10,11,12], including the use of special nanomaterials as platforms for targeted drug delivery [6], therapeutic diagnostic for cancer.

Photothermal therapy (PTT) [13,14], chemodynamic therapy (CDT) [15,16], immunotherapy [17,18,19], and a combination of two or more therapies can reduce the risk of side effects and enhance anti-cancer efficacy [20,21,22,23,24]. The detection of characteristic cancer markers and bioimaging in cancer therapy are important components of current cancer treatment [25,26,27]. In addition, early diagnosis of malignancies based on clinical aspects and in situ monitoring of lesion areas play a key role in reducing the side effects. This highly depends on the contrast agent and the decent use of bioimaging techniques [28]. Since the high spatiotemporal resolution of imaging technology, bioimaging has been proven to be an effective tool for the visualization of biological specimens [29]. Moreover, the visual detection of cells and tissues favors the understanding of the mechanism of diseases [30]. Early diseases can be detected and characterized with appropriate molecular probes or contrast agents [31]. The combination of porous framework materials with bioimaging shows great promise in developing more efficient medical bioimaging systems [32,33,34].

**Scheme 1:**
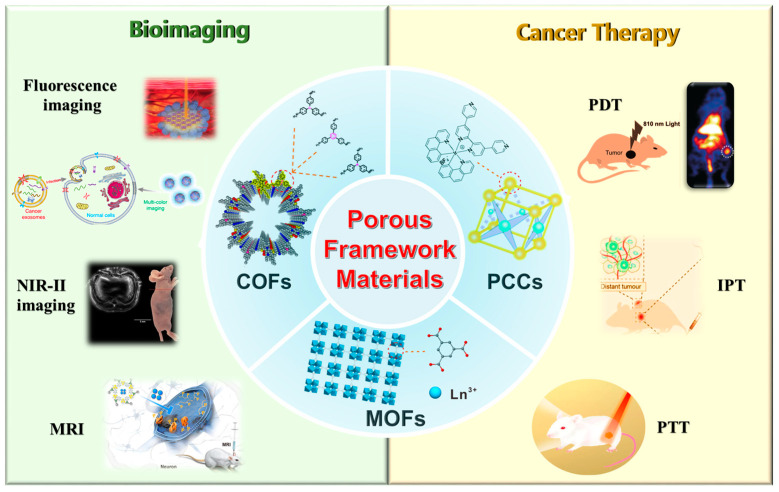
Porous framework materials in bioimaging and cancer therapy.

Herein, we present the recent advances in metal-organic frameworks (MOFs) [35,36,37,38,39,40,41,42,43], covalent organic frameworks (COFs) [44,45,46,47,48,49], and porous coordination cages (PCCs) [50,51,52] for cancer therapy, mainly in bioimaging, and treatments using photothermal therapy, thermodynamic therapy, or a combination of different therapies in cancer therapy (Scheme 1). We highlight the applications of porous framework materials for bioimaging and cancer therapy in this article (Table 1). In addition, we share our point of view on the limitations and future directional aspects regarding porous framework-based nanomaterials in cancer therapy.

## 2. Porous Framework Materials in Bioimaging

As an emerging field, bioimaging is defined as the visualization of biological processes through various probes with modern advanced instrumentations. To date, supramolecular porous framework materials have been extensively investigated in various bioimaging, including fluorescence imaging (FL), magnetic resonance imaging (MRI), computed tomography (CT), and multimodal imaging [28,30,32,85,86,87,88]. Among them, fluorescence imaging has shown higher sensitivity, simpler operating functions, and faster imaging characteristics, and is gaining attention in tumor identification and real-time navigation surgery clinics [3]. Thus, porous frame materials are widely used in fluorescence imaging, including near-infrared biological imaging, upconversion fluorescence imaging, and single/two-photon fluorescence biological imaging (Table 1). Furthermore, nanodiagnostics and therapeutics based on porous framework materials can enable effective and precise diagnosis and treatment of various diseases [89].

### 2.1. MOF in Bioimaging

MOFs have good biocompatibility, drug loading, and biodegradability, which is beneficial to live cell imaging. Also, nanoscale MOF can be easily endocytosed by cells, offering the possibility of cellular imaging [85]. By combining other fluorescent materials, such as organic fluorescent dyes and carbon quantum dots, the ability of MOF to absorb excitation photons can be enhanced, therefore, improving its imaging performance [85,90,91,92]. Another method is to functionalize the surface of MOF and fabricate modified MOF materials to encapsulate drugs [54]. Functional MOF-based contrast agents are beneficial to achieve enhanced fluorescence and targeted localization. As a result, the effect of targeted imaging can be obtained.

**Figure 1:**
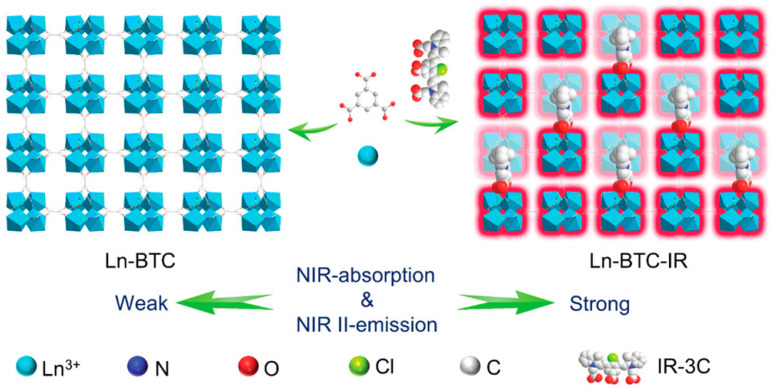
Schematic diagram of the fabrication of **Ln-BTC-MOFs** with strong NIR-II emission by a cyanine dye doping process. Reprinted with permission from [53]. Copyright 2022 John Wiley and Sons.

The wavelength of the second near-infrared region (NIR-II) is located in the range of 1000–1700 nm, enabling a lesser photon scattering and weaker tissue self-fluorescence, which can greatly improve the detection depth, resolution and sensitivity of fluorescence imaging [93]. Thus, NIR-II region fluorescence imaging is a large-depth, high-resolution optical in vivo imaging tool. Liang et al. [53] reported a strategy for obtaining Ln-based MOFs (**Ln-BTC-MOFs**, BTC = 1,3,5-benzenetricarboxylate as ligand; Ln = Yb3+, Nd3+, Er3+) featuring NIR-II/IIb emitting and high luminescence efficiency that can be used for bioimaging. They achieved a significant improvement in NIR-II emission by doping the NIR cyanine dye (IR-3C) into **Ln-BTC-MOFs**, which enhanced the absorption of excitation photons (Figure 1). More importantly, the **Er-BTC-IR@A** modified by amphiphilic molecules exhibits strong near-infrared emission in the aqueous phase and has good biocompatibility, making it suitable for real biological applications. High-resolution NIR-IIb luminescence imaging of lymph, blood vessels, and spine was successfully achieved after intravenous injection of **Er-BTC-IR@A** in mice. This study serves as an inspiration for the development of MOFs in near-infrared imaging and will stimulate more applications of MOF in NIR-II fluorescence imaging.

**Figure 2:**
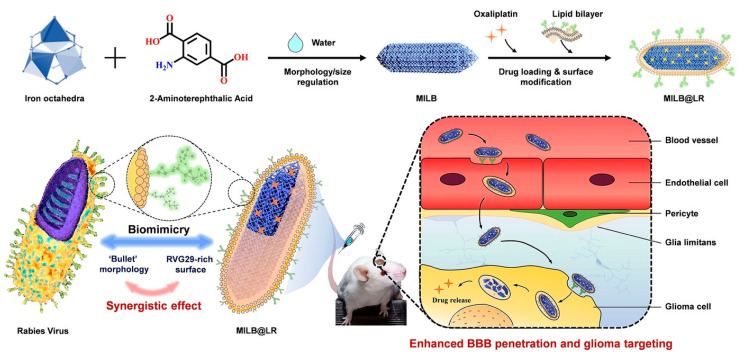
Manufacture of **MILB@LR** and a comprehensive simulation strategy on RABV to enhance BBB infiltration and chemotherapy for glioma. Reprinted with permission from [54]. Copyright 2020 John Wiley and Sons.

Qiao et al. [54] successfully developed a novel targeted drug delivery vector **MILB@LR** for the effective treatment of glioblastoma multiforme (GBM) (Figure 2). MILB was further functionalized with a lipid bilayer composed of part of the RVG29 peptide, that is, **MILB@LR**. Due to the excellent modifiability of MOF, its surface structure can be precisely tuned to highly mimic surface functions of natural rabies virus (RABV) and bullet morphology. Using MOF as a contrast agent, the modified **MILB@LR** can pass through the blood-brain barrier (BBB) and reach the brain tumor cells, achieving the effect of targeted brain tumor cell imaging. **MILB@LR** has good stability and biocompatibility and exhibits excellent BBB penetration and tumor targeting to the brain. This material could be a prospective nanocarrier for the high-efficiency delivery of therapeutic drugs for tumors and other diseases, and this work opens up avenues for the biomedical drug delivery of nanomaterials. 

High-order multiphoton excitation fluorescence (H-MPEF) materials typically exhibit nonlinearity and NIR-II excitation, which has the advantages of high sensitivity, strong penetration depth, and low phototoxicity [94]. Therefore, these materials have great potential in tumor diagnosis and treatment. Recently, the Li group [55] prepared a Zirconium-based MOF (**ZrTc**) using 4,4′- (thiazole[5,4-d]thiazole-2,5-diyl)dibenzoic acid (Tc) as the multiphoton active unit. Functional modification of **ZrTc** surface by ligand interaction to obtain **ZTIG** (Galactose-modified **ZrTc**) with good stability and tumor cell-specific targeting ability. The ordered ligand arrangement in **ZrTc** effectively avoids fluorescence quenching caused by π–π stacking, and the charge transfer from the organic ligand to the metal node can further improve electron delocalization, thus, improving its performance. In addition, **ZTIG** exhibits cancer cell-specific targeting ability through coordination effects between Zr clusters and O atoms. More importantly, its fluorescence imaging with high spatial resolution and deep tissue penetration can be achieved under light excitation in the near-infrared region II, making it a potential material for cancer diagnosis. This work represents the first reported MOF-based H-MPEF material for fluorescence imaging with conceptual applications in cancer diagnosis and therapy.

Recent years have witnessed rapid development in the applications of MOFs in bioimaging. Outstanding features of MOFs, such as porous morphology, high porosity, adjustable building blocks, targeting ability, and biological stability, have contributed to their progress in bioimaging.

### 2.2. COF in Bioimaging

COFs have been increasingly used in bioanalysis and nanomedicine in recent years due to their porous structure and ultra-high specific surface area, as well as excellent photostability and non-cytotoxicity [64,87]. The precise structure of COFs can be determined by powder x-ray diffraction (XRD), although obtaining their single crystal structure remains extremely difficult. The extended long-range ordered structures of COFs endow them with excellent broad-spectrum light absorption, suggestive of great potential in collecting and quenching fluorescence from a variety of dyes. Moreover, modified COF materials, in which functional nanomolecules serve as probes and the COFs themselves are used for fluorescence imaging, facilitate localization detection by tracking the location of the probes [62]. This is of great significance for accurate analysis. Some COFs are directly bound to fluorescent probes, whose fluorescence imaging effect can be enhanced using a two-photon fluorescence imaging technique [61].

**Figure 3:**
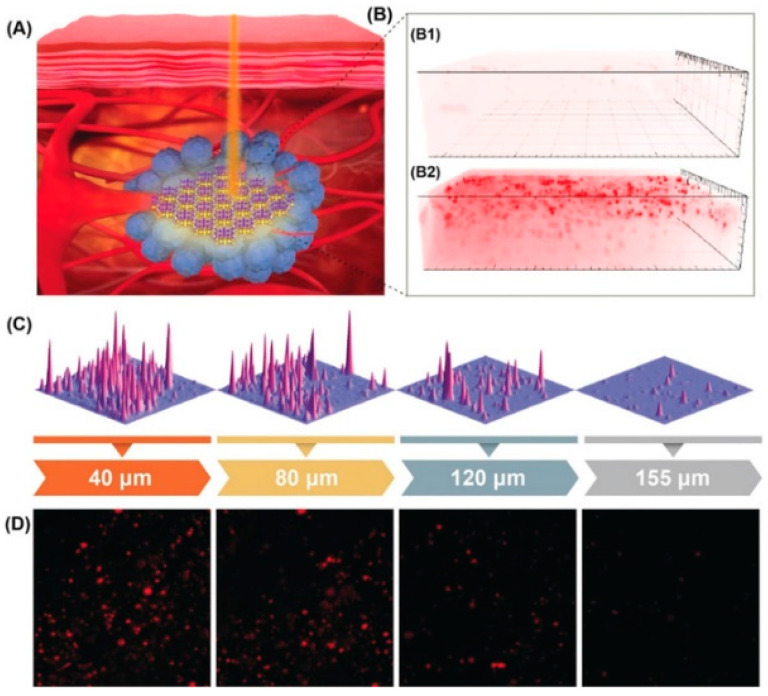
(**A**) Schematic diagram of **TPI-COF** for in vivo tumor imaging. (**B**) The two-photon induced fluorescence imaging study of **TPI-COF** in Balb/c mice model with 4T1 tumor xenograft (B1, PBS group; B2, **TPI-COF** group). (**C**) The two-photon fluorescence intensity of **TPI-COF** at different tumor tissue depths. (**D**) The two-photon fluorescence imaging of **TPI-COF** at different tumor tissue depths. Reprinted with permission from [61]. Copyright 2019 John Wiley and Sons.

In 2019, Zeng et al. [61] prepared a benzothiadiazole-based covalent organic framework through a simple solvothermal method. This COF (**TPI-COF**) can promote two-photon induction (TPI), exhibiting efficient TPI fluorescence emission. They further established a Balb/c mouse 4T1 tumor xenograft model to verify the feasibility of **TPI-COF** in vivo. Two-photon fluorescence imaging of mice after intravenous injection of **TPI-COF**, measured by two-photon electrophysiological microscopy (Figure 3A). A distinct punctate fluorescence signal was observed in the tumor tissue under 810 nm TPI (Figure 3B). Figure 3C,D shows that the maximum detectable depth of **TPI-COF** is up to about 150 μm. It uncovered the potential of **TPI-COF** as a two-photon probe in studying biological cells and tissues. This study will provide direction for future biomedical applications of near-infrared **TPI-COFs**.

Hydrogen sulfide (H_2_S) plays a crucial role in the physiology of the human body. Abnormal levels of H_2_S in cells are associated with major diseases such as cancer, which makes it a potential target for cancer therapy [95]. In 2018, Zhang et al. [62] proposed to first create a hybrid COF-based probe and extended an effective anti-interference strategy based on it. As a proof-of-concept, they chose 4-amino-1,8-naphthalimide derivative (NPHS) as two-photon fluorescent probes, which have a large two-photon absorption cross-section. Then, they fabricated a COF nanoprobe with two-photon fluorescence by p-toluenesulfonic acid-mediated solvothermal method, **TpASH-NPHS**, for targeting hydrogen sulfide as a model analyte. Compared with the small molecule probes, the **TpASH-NPHS COF** exhibits high H_2_S selectivity and low cytotoxicity and can be used for fluorescence imaging in deep tumor tissues. In addition, the assay process is not compromised by casual interactions with endogenous enzymes, which is verified by preliminary results in a mouse model of cirrhosis. Such fluorescent COF nanoprobes hold great prospects in terms of bioimaging and nanomedicine research.

**Figure 4:**
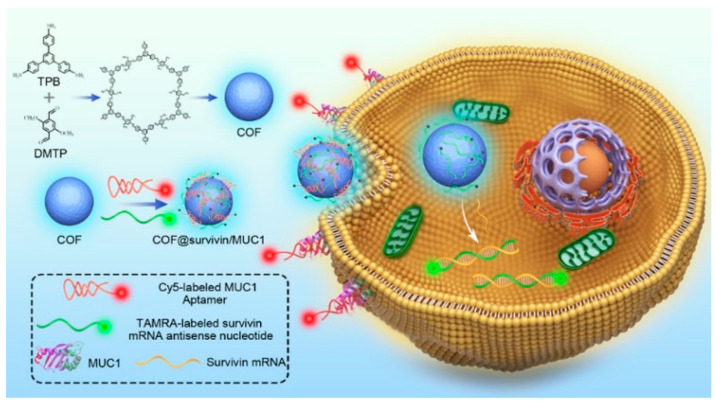
Synthesis of COF and the multicolor nanoprobe, and its schematic application in cell imaging. Reprinted with permission from [63]. Copyright 2021 American Chemical Society.

The spatial distribution of different biomarkers in cancer cells varies, and monitoring the expression and localization of different biomarkers in living cells can improve cancer diagnosis. In 2021, the Tang group [63] fabricated a COF-based tricolor fluorescent nanoprobe (**COF@survivin/MUC1**) by a freezing method and applied it to the simultaneous imaging of biomarkers with different distributions in living cells (Figure 4). **COF@survivin/MUC1** is composed of COF nanoparticles adsorbing a Cy5-labeled aptamer MUC1 and a TAMRA-labeled survivin mRNA antisense oligonucleotide. In contrast to the existing nanoprobes, the intrinsic fluorescence of this material can monitor the distribution of nanoprobes inside and outside the cell. Accordingly, biomarkers with different spatial distributions were successfully visualized using nanoprobes. This work provides new insights into bioimaging and biomarker detection based on COF nanoprobes.

Overall, COF possesses several significant advantages in biomedical applications. First, the large pore volume of COFs enables high drug and probes loading capacity; second, their reversible covalent bonds enable a better biodegradability range; third, the high π electron density of COFs provides excellent performance for bioimaging.

### 2.3. PCC in Bioimaging

In recent years, remarkable development has been achieved in the synthesis and structure determination of PCCs. They have also attracted attention as molecular containers for biomedical applications such as drug delivery, bioimaging, and cancer therapy. However, reports on the applications of PCCs in bioimaging remain rare, and the main focus is on enhancing fluorescence imaging by utilizing the fluorescence properties of ligand groups [66]. Porous organic cages (POCs), which are similar to PCCs but do not contain metals, are also a potential material for bioimaging [67].

**Figure 5:**
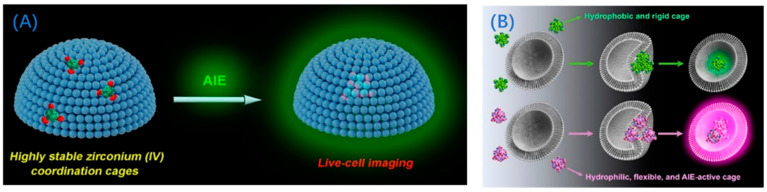
(**A**) Schematic diagram of the application of self-assembly of a highly stable Zirconium(IV) coordination cages containing AIE molecular rotors for live cell imaging (red color groups: the AIE molecular rotors). (**B**) AIE-active POCs with hydrophilic and stimuli-responsive properties for bioimaging enhancement. Reprinted with permission from [68]. Copyright 2020 John Wiley and Sons. Reprinted with permission from [66]. Copyright 2022 American Chemical Society.

In 2019, the Zhao group [68] reported an assembly consisting of structurally stable Zirconium(IV)-based coordination cages and aggregation-induced emission (AIE) molecular rotors (Figure 5A). Then it was applied to bioimaging in vitro. The results show that the fluorescence emission intensity of the cage can be effectively controlled by limiting the kinetic behavior of the AIE molecular rotor. Since coordination cages have excellent chemical stability in aqueous solution and the molecular rotor features good AIE properties, they can be employed as novel fluorescent probes to image living cells in vitro.

There are also some cases of POCs being used in biomedicine. For example, POCs can be used as effective imaging agents to enhance fluorescence imaging and have low cytotoxicity. Zhao et al. [66] used a POC with AIE activity and a stimulus-responsive capability to realize bioimaging (Figure 5B). Notably, the imine bonds in the coordination unit are completely transformed into amine bonds under mild conditions, which greatly improves its hydrophilicity and the efficiency of living cell imaging. Dana Al Kelabi et al. [67] reported a reciprocal heterotrimeric organic cage with biological activity and mitochondrial targeting. They demonstrated that a biocompatible organic cage (OC1) can be employed as a mitochondria-targeted fluorescent probe with high cell permeability and impressive photostability. Notably, its stability and fluorescence are better than some commercial materials. These examples demonstrate the potential of POCs with good biocompatibility in biomedicine, although their real-world applications remain challenging.

As two discrete porous framework materials, PCCs and POCs hold great promise for bioimaging due to their good solubility and excellent fluorescence tunability.

## 3. Porous Framework Materials for Cancer Therapy

MOFs, COFs, and PCCs can integrate different nanoparticles or biomolecules into their frameworks due to their size tunability and structural diversity. It is beneficial to improve the loading efficiency of guest molecules and contributes to the formation of nanocarriers with excellent biocompatibility, water solubility, and biodegradability [50]. Therefore, they can be applied to customizable therapeutic diagnostic platforms for cancer therapy, including photodynamic therapy (PDT), photothermal therapy (PTT), chemodynamic therapy (CDT), and combination therapy (Table 1).

### 3.1. MOF in Cancer Therapy

It is well known that MOF materials are extensively used in PTT, PDT, CDT, and combination therapy. The functionalization of MOFs enables them to effectively target cancer cells [69]. Encapsulation of photosensitizer (**Ps**) into MOFs can avoid the aggregation of **Ps** and improve the ability of the composite to generate reactive oxygen species (ROS) upon light irradiation. Such a composite material can be used for PDT [70]. Moreover, Fe-MOFs loaded with organic materials are usually used for CDT, which can induce iron death in tumor cells [76]. Composite materials such as NPs@MOFs [96] and biomolecules@MOF [97] are also excellent candidates for cancer treatment.

**Figure 6:**
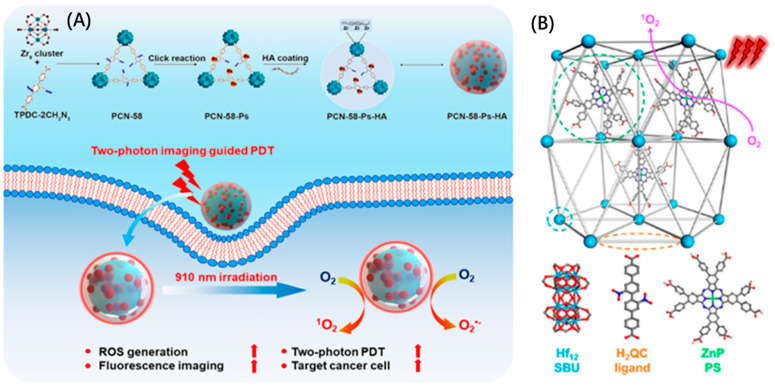
(**A**) Preparation of **PCN-58-Ps-HA** and highlighting of photo-induced ROS generation and two-photon fluorescence imaging-guided PDT. (**B**) Structural composition of **ZnP@Hf-QC**: a 3D framework of Hf_12_ SBUs and QC bridging ligands and ZnP PSs confined in the pore space. **ZnP@Hf-QC** efficiently produced ^1^O_2_ under light irradiation at 700 nm. Reprinted with permission from [69,70]. Copyright 2021 American Chemical Society.

The therapeutic effect of PDT mainly depends on the ability of the **Ps** to generate ROS upon light irradiation. The Li group [69] synthesized a two-photon-active metal−organic framework **PCN-58-Ps** by a click reaction. Then a functionalized **PCN-58-Ps**-**HA** was obtained by capping **PCN-58-Ps** with hyaluronic acid (HA) through a coordination effect (Figure 6A), whose pore channels effectively prevented Ps aggregation. They found that the ROS generation of **PCN-58-Ps** is related to the inter-systemic crossover (ISC) of **Ps** and the linker-to-cluster charge transfer (LCCT) of **PCN-58**. Upon the NIR laser irradiation at 910 nm, **PCN-58-Ps-HA** with two-photon activity enables generate abundant ROS (^1^O_2_ and O_2_•^-^), which is a key factor for cell apoptosis.

Lin et al. [70] constructed a **Hf-QC** nanoscale metal−organic framework (nMOF) based on Hf_12_ and 2″,3′-dinitro-[1,1′:4′,1″;4″,1‴-quaterphenyl]-4,4‴-dicarboxylate (QC), and then encapsulated zinc-phthalocyanine (ZnP) into the pores of the rigid **Hf-QC** backbone to prepare **ZnP@Hf-QC**. Such a composite material avoids ZnP aggregation-induced excited state bursts and significantly enhances ROS generation upon light irradiation (Figure 6B). Importantly, its mediated PDT has better biocompatibility. Experimental results proved that ultra-high tumor growth inhibition and cure rates in mouse colon cancer models can be achieved, with excellent anti-tumor efficacy.

Monotherapy for tumor treatment has poor clinical efficacy due to the limitation of response conditions and the emergence of multidrug-resistant bacteria. Therefore, the construction of CDT- and PTT-based combination therapy is of great social significance.

Shen et al. [76] reported a nano-sized MOF containing piperlongumine (PL) modified by polydopamine (PDA) and new indocyanine green (IR820), that is, **MP@PI**. They found that MOF and PL were utilized as the iron source and H_2_O_2_ source, respectively, for CDT. Moreover, MOF and PL can promote iron death in tumor cells, which further leads to ROS production, lipid peroxide (LPO) accumulation, and glutathione peroxidase 4 (GPX4) downregulation. The nano-platform, assisted by PTT, was used to induce an in-vivo immune response, enabling synergistic anti-tumor effects in-vitro and in-vivo.

The combination of MOFs and functional materials has led to the creation of novel multifunctional composites for cancer therapy. They have also shown great potential in targeted and multimodal therapy for improving cancer treatment efficiency [98].

### 3.2. COF in Cancer Therapy

COFs are generally composed of aromatic molecules, which make their absorption spectra lie mainly in the high-energy range of 320–450 nm [99]. In addition, organic backbones bearing electron-donating structural units (donors) and electron-accepting structural units (acceptors) are usually developed as photothermal agents for PTT.

The donor and acceptor in COF materials convert the absorbed light energy into heat energy through the occurrence of photo-induced electron transfer, which means that the COF material acts as a photothermal agent releasing heat through non-radiative relaxation, thus, leading to tumor ablation. The maximum absorption wavelength of COFs can be modulated by optimizing the structure of the donor to be suitable for cancer therapy [78]. The biocompatibility of COFs can also be improved by wrapping polysaccharides on their surface [79]. COFs can be excellent carriers of hydrophobic drugs by adjusting their pore size [100].

**Figure 7:**
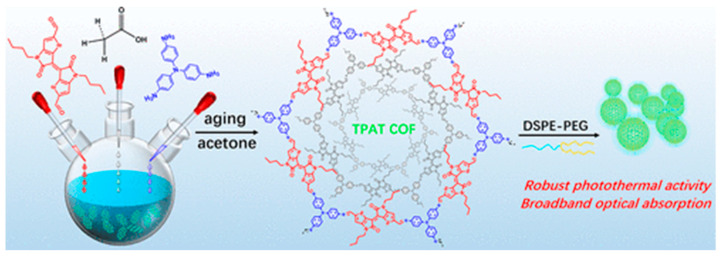
Schematic diagram of the synthesis of **TPAT COF** and its photothermal application to tumors. Reprinted with permission from ref [77]. Copyright 2022 American Chemical Society.

The Xie group [77] synthesized a novel COF with adjustable size and strong colloidal stability through Schiff base reaction in acetone at room temperature, which is constructed by electron-deficient thiophene isoindigo and electron-rich triphenylamine (**TPAT COF**). **TPAT COF** exhibits broad absorption even in the NIR-II due to the intramolecular charge transfer effect combined with the extension of the π–π conjugated backbone of the COF (Figure 7). Moreover, this COF possesses a high photothermal conversion efficiency (PCE) under 808 nm laser irradiation, allowing it to exhibit a significant cancer cell-killing effect.

**Figure 8:**
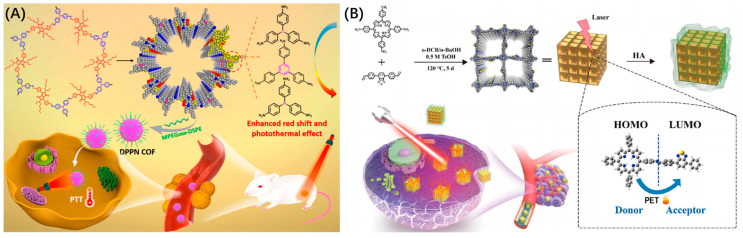
(**A**) Schematic synthesis of D-A structured COFs. (**B**) Schematic design and preparation of **TB-COF-HA** for PTT. Reprinted with permission from [78]. Copyright 2021 American Chemical Society. Reprinted with permission from [79]. Copyright 2022 Springer Nature.

Based on the same donor-acceptor strategy, the Jing group [78] also obtained a series of size-controlled COFs by Schiff base reaction under mild conditions. Interestingly, the COFs modified by 1,2-distearoyl-sn-glycero-3-phosphoethanolamine-N-[methoxy (polyethylene glycol)] (MPEG 2000-DSPE) show excellent colloidal stability and biocompatibility in water (Figure 8A). Moreover, these COFs exhibit a broad absorption spectrum with a maximum absorption wavelength that can be tuned by optimizing the structure of the donor and can even cover the NIR-II biological window for effective tumor growth inhibition under laser irradiation.

Tang et al. [79] use electron-rich 4,4′,4″,4‴-[porphyrin-5,10,15,20-tetrayl]tetraaniline (TAPP) and electron-deficient 4,4′-[benzothiadiazole-4,7-diyl]- dibenzaldehyde (BDA) to construct **TB-COF**. Remarkable photo-induced electron transfer (PET) occurs between the donor and acceptor in **TB-COF** under laser irradiation, which converts the absorbed light energy into heat energy, leading to tumor ablation. Its photothermal conversion efficiency is comparable with that of the commonly used PTT material (Figure 8B). In addition, wrapping a layer of HA on the surface of **TB-COF** (**TPA-COF-HA**) greatly improves its biocompatibility and enables tumor targeting, thus, enhancing therapeutic efficacy and drug safety.

The COFs contain both donors and acceptors, allowing charge-transfer jumps to occur at lower energies, greatly improving their optical properties and, thus, enabling effective photothermal treatment of tumors under laser irradiation [101].

### 3.3. PCC in Cancer Therapy

PCCs can also be used in PDT treatment due to their excellent ability to produce highly reactive singlet oxygen (^1^O_2_) [102]. On the one hand, **Ps** can be introduced to construct cages to promote the production of ROS [84]. On the other hand, doping heavy atoms into PCCs can produce high-yield ROS for PDT enhancement [103].

In cancer therapy, single molecule nanoparticles (SMNPs) can combine both imaging and therapeutic capabilities and show unparalleled advantages. Therefore, Chen’s group [83] used a template-directed strategy to synthesize a porphyrin nanocage, which is further constructed to **porSMNPs** by post-polyethylene glycol chain modification. Such functionalized **porSMNPs** can be employed as a therapeutic diagnostic platform to monitor the whole process of drug metabolism using radioactive ^64^Cu-labeled imaging. In addition, the cage-like structure significantly improves the photosensitization of **porSMNPs** by inhibiting π–π stacking interactions of **Ps**, which in turn enhances the antitumor performance in PDT.

**Figure 9:**
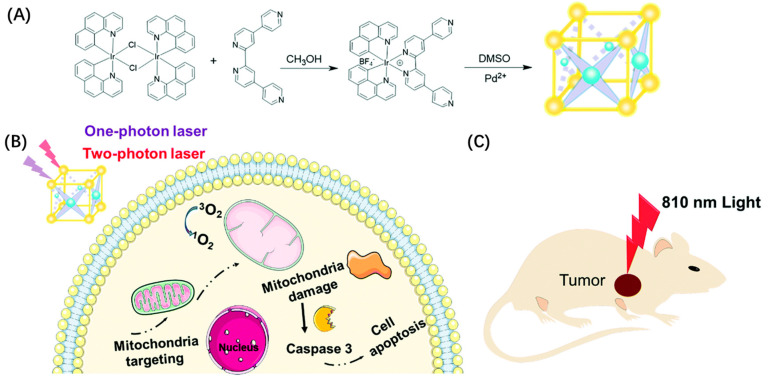
(**A**) Synthesis of **MOC-53**. (**B**,**C**) Schematic diagram of the PDT effect of **MOC-53** on a tumor-bearing mouse under 810 nm light irritation. Reprinted with permission from [84]. Copyright 2022 Royal Society of Chemistry.

Su et al. [84] prepared a photoactive highly ordered cubic [Pd_4_Ir_8_]^16+^ cage (**MOC-53**), which is composed of eight Ir(III)-based **Ps** and four Pd-based receptors (Figure 9). Such cage material was used as an efficient mitochondria-targeting single- and two-photon PDT reagent. **MOC-53** has excellent ^1^O_2_ production efficiency, cellular uptake ability, and specific mitochondrial targeting capacity, leading to effective cancer cell death.

It is urgent to develop PCCs with good light absorption as ideal Ps that can not only target mitochondria to create an energy crisis but also generate a high yield of ROS leading to apoptosis and, thus, improve the efficacy of photodynamic therapy [104].

## 4. Conclusions, Challenges, and Perspectives

Great advances have been achieved in the synthesis and various applications of porous framework materials (i.e., MOFs, COFs, and PCCs) over the past two decades, including catalysis, biomedicine, and sense. Here we summarize the progress and advantages of these materials in bioimaging and cancer treatment. For example, the optical properties of porous framework materials can be easily modulated by controlling their component and structure to achieve excellent performance on bioimaging. The use of porous framework materials for bioimaging can, to a certain extent, solve the problem of cancer diagnosis at the primary stage. More importantly, their precise structure favors the investigation of the “structure-performance relationship”. Furthermore, their porosity is a unique advantage in improving drug-carrying capacity, showing promising potential in cancer treatment.

Even so, it is still at an early stage of development for their application in cancer therapy. There are still several important issues that need to be solved regarding fundamental research and medical applications of porous materials. First, current research in this field mainly focused on the uptake of nanomaterials in cells and tumors, rather than their metabolism and whereabouts. It is important to elucidate the in-vitro and in-vivo metabolic pathways of porous framework materials. This requires rapid degradation and in-vivo clearance of porous framework materials. Second, the biological toxicity of nanomaterials also needs to be evaluated in a long-term and more in-depth manner. The more stable nanomaterials are, the more difficult they may be to degrade, which has implications for their high biosafety. Third, it is still difficult to fully understand the effect of structure on performance in bioimaging and cancer therapy. Finally, activation of the immune response through porous framework materials has still not achieved satisfactory results in cancer therapy.

Therefore, future efforts should focus on the rational structural design of porous framework materials involving the selection of metal ions and ligands with hypotoxicity, and their stability in biological environments. In addition, more functions applicable to the tumor microenvironment, such as efficient drug tracking, should be added to the design. Since the fluorescence properties of most materials are readily influenced in the biological environment, it is necessary to design and develop excellent fluorescent materials with good stability suitable for in vivo imaging as probes.

As discrete porous materials, PCCs typically contain metal, vertex ligands, and plane ligands, and possess a unique geometric structure (e.g., tetrahedron and octahedron). Numerous porous coordination cages have been synthesized up to now, and their cavity size, solubility, and topology are readily tuned by ligands. In particular, PCCs with the same topology but different metals provide an ideal platform for investigating the role of the components on performance. In addition, functionalizing vertex ligands to construct water-soluble PCCs would be a promising direction to achieve biological applications. More importantly, the fluorescence properties of PCCs can be fine-tuned by modifying plane ligands or metal species to improve their performance in biological imaging. However, the unsatisfactory stability of PCCs limits their practical application to some extent. It is a promising direction to explore ultra-stable PCCs through a chelation assembly strategy.

Traditional therapies such as radiotherapy and chemotherapy primarily attack cells indiscriminately. In contrast, techniques including PTT and PDT are largely site-specific but sometimes not as effective. Consequently, it deserves to combine new strategies based on porous framework materials with traditional methods for achieving better performance in cancer treatment, and the future effort will focus on the eradication of cancer cells to reduce the risk of recurrence.

Despite various challenges with respect to porous framework materials for cancer therapy, it is believed that nanomedicine cancer therapy based on these materials is a promising growth area.

## Figures and Tables

**Table 1:** Recent advances in porous framework materials for bioimaging and cancer therapy.

Applications		Porous Framework Materials		Main Functions	Ref.
Bioimaging	NIR-Ⅱ imaging	MOFs	Ln-BTC-MOF (Ln = Yb^3+^, Nd^3+^, Er^3+^)	enhanced the absorption of excitation photons	[53]
	fluorescence imaging		MIL-Fe	as a contrast agent	[54]
	NIR-Ⅱ imaging		ZrTc; ZrTcl	activated H-MPEF performance	[55]
	magnetic resonance imaging (MRI)		MIL-101(Fe)	high spatial resolution and deep penetration	[56]
	multimode bioimaging		ICG-CpG@MOF	comprehensive diagnostic information	[57]
	MRI		Fe-MIL-88B-NH_2_-NOTA-DMK6_240_/MB	enhance tau targeting	[58]
	fluorescence imaging		PCN-CuS-FA-ICG	facile diffusion of ^1^O_2_ and prevention of PS self-quenching	[59]
	MRI		FDGI NPs	could effectively accumulate FDG (Fe-DOX@Gd-MOF) NPs at the tumor site	[60]
	two-photon fluorescence imaging	COFs	TPI-COF	improves the delocalization of π electrons, and leads to a high dipole value and fluorescent activity	[61]
	two-photon fluorescence imaging		TpASH-NPHS	minimize cellular autofluorescence, reduce tissue injury and increase tissue penetration depth	[62]
	fluorescence imaging		COF@survivin/MUC1	employed a freezing method to improve the DNA loading density and ensure detection performance	[63]
	Multicolor imaging		Pd NPs/CMC− COF-LZU1	produce obvious changes in both color and fluorescence	[64]
	bioimaging		TTA-DFP CONs	possess tunable optical characteristics	[65]
	fluorescence imaging	PCCs	Zr_6_L_3_ coordination cage	good aggregation-induced emission (AIE) properties	[66]
	fluorescence imaging		TPE-cage	good aggregation-induced emission (AIE) properties	[67]
	fluorescence imaging		POC-OC1	high biocompatibility, cell permeability, and mitochondrial targetability	[68]
Cancer therapy	PDT	MOFs	PCN-58-Ps-HA	generates abundant reactive oxygen species (ROS)	[69]
	PDT		ZnP@Hf-QC	significantly enhances ROS generation upon light irradiation	[70]
	PDT		Au@MOF	leads to ROS production	[71]
	CDT		Cu(bpy)_2_(OTf)_2_	facilitates Fenton-like reactions	[72]
	PTT+PDT		HUC-PEG	leads to ROS production	[73]
	CDT+PDT		FeTCPP/Fe_2_O_3_ MOF	leads to ROS and ·OH production	[74]
	Chemotherapy+CDT		TA-MOF	leads to ·OH production and facilitates Fenton reactions	[75]
	CDT+PTT		MP@PI	promote iron death and leads to ROS production	[76]
	PTT	COFs	TPAT COF	possesses a high photothermal conversion efficiency (PCE) under 808 nm	[77]
	PTT		DPPN COF	possess potent photothermal activity under laser irradiation	[78]
	PTT		TB-COF	the donor and acceptor under laser irradiation and convert the absorbed light energy into heat energy	[79]
	PDT		PCPP	leads to ROS production	[80]
	PDT		UCCOFs	leads to ROS production	[81]
	IPT (immunogenic photo-therapy)		ICG@COF-1@ PDA	enhanced photodynamic and photothermal therapy	[82]
	PDT	PCCs	porSMNPs	inhibit π–π stacking interactions of photosensitizers and enhance the antitumor performance	[83]
	PDT		MOC-53	excellent ^1^O_2_ production efficiency, cellular uptake ability, and specific mitochondrial targeting capacity	[84]

## Data Availability

No applicable.
